# Propane-1,3-diaminium bis­(perchlorate)–18-crown-6 (1/2)

**DOI:** 10.1107/S1600536811056248

**Published:** 2012-01-11

**Authors:** Min-Min Zhao

**Affiliations:** a Ordered Matter Science Research Center, College of Chemistry and Chemical Engineering, Southeast University, Nanjing 210096, People’s Republic of China

## Abstract

In the title compound, C_3_H_12_N_2_^2+^·2ClO_4_^−^·2C_12_H_24_O_6_, the central C atom of the propane-1,3-diammonium cation is located on a twofold rotation axis and the two terminal –NH_3_ groups insert into the crown rings through N—H⋯O hydrogen bonding, resulting in the formation of a 1:2 supra­molecular [(C_3_H_12_N_2_)·(C_12_H_24_O_6_)_2_]^+^ complex. The perchlorate anion links with the supra­molecular complex *via* weak C—H⋯O hydrogen bonding.

## Related literature

For the properties and structures of related compounds, see: Fu *et al.* (2007 ▶, 2008 ▶, 2009 ▶); Fu & Xiong (2008 ▶). For the ferroelectric properties of related amino derivatives, see: Fu *et al.* (2011*a* ▶,*b* ▶,*c* ▶).
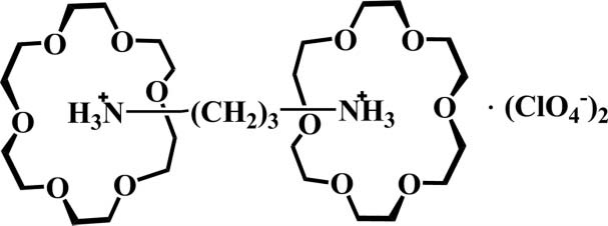


## Experimental

### 

#### Crystal data

C_3_H_12_N_2_^2+^·2ClO_4_^−^·2C_12_H_24_O_6_
*M* *_r_* = 803.67
Monoclinic, 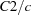

*a* = 22.984 (5) Å
*b* = 9.0055 (18) Å
*c* = 21.620 (4) Å
β = 113.59 (3)°
*V* = 4101.0 (17) Å^3^
*Z* = 4
Mo *K*α radiation
μ = 0.23 mm^−1^
*T* = 298 K
0.10 × 0.03 × 0.03 mm

#### Data collection

Rigaku Mercury2 diffractometer
Absorption correction: multi-scan (*CrystalClear*; Rigaku, 2005 ▶) *T*_min_ = 0.910, *T*_max_ = 1.000
16855 measured reflections
3606 independent reflections
2059 reflections with *I* > 2σ(*I*)
*R*_int_ = 0.086

#### Refinement

*R*[*F* ^2^ > 2σ(*F* ^2^)] = 0.085
*wR*(*F* ^2^) = 0.248
*S* = 1.07
3606 reflections
232 parameters
H-atom parameters constrained
Δρ_max_ = 0.65 e Å^−3^
Δρ_min_ = −0.42 e Å^−3^

### 

Data collection: *CrystalClear* (Rigaku, 2005 ▶); cell refinement: *CrystalClear*; data reduction: *CrystalClear*; program(s) used to solve structure: *SHELXS97* (Sheldrick, 2008 ▶); program(s) used to refine structure: *SHELXL97* (Sheldrick, 2008 ▶); molecular graphics: *SHELXTL* (Sheldrick, 2008 ▶); software used to prepare material for publication: *SHELXTL*.

## Supplementary Material

Crystal structure: contains datablock(s) I, global. DOI: 10.1107/S1600536811056248/xu5433sup1.cif

Structure factors: contains datablock(s) I. DOI: 10.1107/S1600536811056248/xu5433Isup2.hkl

Additional supplementary materials:  crystallographic information; 3D view; checkCIF report

## Figures and Tables

**Table 1:** Hydrogen-bond geometry (Å, °)

*D*—H⋯*A*	*D*—H	H⋯*A*	*D*⋯*A*	*D*—H⋯*A*
N1—H1*C*⋯O1	0.89	2.10	2.958 (4)	161
N1—H1*E*⋯O3	0.89	2.08	2.951 (5)	167
N1—H1*D*⋯O5	0.89	2.12	3.007 (5)	177
C10—H10*B*⋯O10	0.96	2.55	3.478 (13)	161
C13—H13*A*⋯O9	0.96	2.58	3.470 (7)	154

**Fig. 1.**
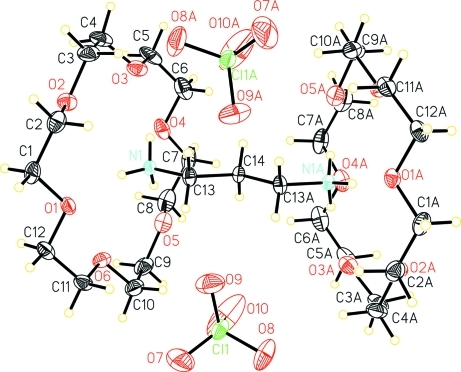
A view of the title compound with the atomic numbering scheme. Displacement ellipsoids are drawn at the 30% probability level.

**Fig. 2.**
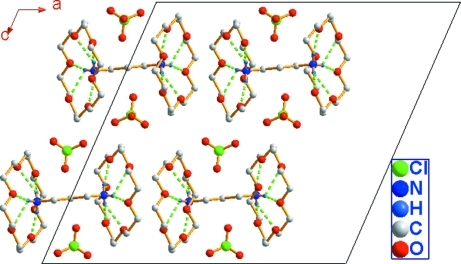
The crystal packing of the title compound, showing the H-bonding interactions. H atoms not involved in hydrogen bonding (dashed line) have been omitted for clarity.

## supplementary crystallographic information

### Comment

Organic amino compounds attracted more attention as phase transition dielectric materials for its application in memory storage (Fu *et al.*, 2007; Fu & Xiong, 2008; Fu *et al.*, 2008; Fu *et al.*, 2009). With the purpose of obtaining phase transition crystals of amino compounds, various amines have been studied and we have elaborated a series of new materials with this organic molecules (Fu *et al.*, 2011*a*; Fu *et al.*, 2011*b*; Fu *et al.*, 2011*c*). In this study, we describe the crystal structure of the title compound, *bis*(18-crown-6)propane-1,3-diammonium perchlorate .

The title compound was composed of cationic [(C_3_H_12_N_2_).(C_12_H_24_O_6_)_2_]^+^ and one ClO_4_^-^ anion (Fig.1). Supramolecular rotators was assembled between protonated propane-1,3-diammonium (H_3_N-C_3_H_6_-NH_3_)^+^ and 18-crown-6 by H-bonds. The ammonium moieties of -NH_3_^+^ cations were interacted with the O atoms of crown ethers through six N—H···O hydrogen bonds, forming a 1:2 supramolecular rotator-stator structures.

The macrocycle adopts a conformation with approximate D_3d_ symmetry, with all O-C-C-O torsion angles being *gauche* and alternating in sign, and all C-O-C-C torsion angles being *trans*. The C-N bonds of cation were almost perpendicular to the mean oxygen planes of crown ethers.

Supramolecular cation structure, [(C_3_H_12_N_2_).(C_12_H_24_O_6_)_2_]^+^, was introduced as counter cation to ClO_4_^-^ anion. Cl has a flattened tetrahedral coordination by four O atoms [range of *cis*-bond angles = 105.2 (5)-113.7 (3) °; dav(Cl-O) = 1.332 (7)-1.410 (4)Å].

The title compound was stabilized by intermolecular N—H···O hydrogen bonds, the ClO_4_^-^ anion not participating in the H-bonding interactions. The intermolecular N—H···O H-bonding length are within the usual range of 2.951 (5) to 3.152 (5)Å. (Table 1 and Fig.2).

### Experimental

The commercial 18-crown-6 (6 mmol), HClO_4_ (6 mmol) and organic amine (3 mmol) were dissolved in water/EtOH (1:1 *v*/*v*) solution. The solvent was slowly evaporated in air affording colourless block-shaped crystals of the title compound suitable for X-ray analysis.

The dielectric constant of title compound as a function of temperature indicates that the permittivity is basically temperature-independent, suggesting that this compound should be not a real ferroelectrics or there may be no distinct phase transition occurred within the measured temperature range. Similarly, below the melting point (405 K) of the compound, the dielectric constant as a function of temperature also goes smoothly, and there is no dielectric anomaly observed (dielectric constant ranging from 4.2 to 7.5).

### Refinement

All H atoms attached to C atoms were fixed geometrically and treated as riding with 0.97 Å (C-methylene). The positional parameters of the H atoms (N1) were initially refined freely, subsequently restrained using a distance of N–H = 0.89 (2) Å, and in the final refinements treated in riding motion of their parent nitrogen atom with *U_iso_*(H)=1.5*U_eq_*(N).

### Crystal data

**Table:**

C_3_H_12_N_2_^2+^·2ClO_4_^−^·2C_12_H_24_O_6_	*F*(000) = 1720
*M**_r_* = 803.67	*D*_x_ = 1.302 Mg m^−^^3^
Monoclinic, *C*2/*c*	Mo *K*α radiation, λ = 0.71073 Å
Hall symbol: -C 2yc	Cell parameters from 3606 reflections
*a* = 22.984 (5) Å	θ = 3.3–27.5°
*b* = 9.0055 (18) Å	µ = 0.23 mm^−^^1^
*c* = 21.620 (4) Å	*T* = 298 K
β = 113.59 (3)°	Block, colorless
*V* = 4101.0 (17) Å^3^	0.10 × 0.03 × 0.03 mm
*Z* = 4	

### Data collection

**Table:**

Rigaku Mercury2 diffractometer	3606 independent reflections
Radiation source: fine-focus sealed tube	2059 reflections with *I* > 2σ(*I*)
graphite	*R*_int_ = 0.086
Detector resolution: 13.6612 pixels mm^-1^	θ_max_ = 25.0°, θ_min_ = 3.3°
CCD profile fitting scans	*h* = −27→27
Absorption correction: multi-scan (*CrystalClear*; Rigaku, 2005)	*k* = −10→10
*T*_min_ = 0.910, *T*_max_ = 1.000	*l* = −25→25
16855 measured reflections	

### Refinement

**Table:**

Refinement on *F*^2^	Primary atom site location: structure-invariant direct methods
Least-squares matrix: full	Secondary atom site location: difference Fourier map
*R*[*F*^2^ > 2σ(*F*^2^)] = 0.085	Hydrogen site location: inferred from neighbouring sites
*wR*(*F*^2^) = 0.248	H-atom parameters constrained
*S* = 1.07	*w* = 1/[σ^2^(*F*_o_^2^) + (0.1136*P*)^2^ + 6.3277*P*] where *P* = (*F*_o_^2^ + 2*F*_c_^2^)/3
3606 reflections	(Δ/σ)_max_ < 0.001
232 parameters	Δρ_max_ = 0.65 e Å^−^^3^
0 restraints	Δρ_min_ = −0.42 e Å^−^^3^

### Special details

**Table:**

Geometry. All esds (except the esd in the dihedral angle between two l.s. planes) are estimated using the full covariance matrix. The cell esds are taken into account individually in the estimation of esds in distances, angles and torsion angles; correlations between esds in cell parameters are only used when they are defined by crystal symmetry. An approximate (isotropic) treatment of cell esds is used for estimating esds involving l.s. planes.
Refinement. Refinement of F^2^ against ALL reflections. The weighted R-factor wR and goodness of fit S are based on F^2^, conventional R-factors R are based on F, with F set to zero for negative F^2^. The threshold expression of F^2^ > 2sigma(F^2^) is used only for calculating R-factors(gt) etc. and is not relevant to the choice of reflections for refinement. R-factors based on F^2^ are statistically about twice as large as those based on F, and R- factors based on ALL data will be even larger.

### Fractional atomic coordinates and isotropic or equivalent isotropic displacement parameters (Å^2^)

**Table:**

	*x*	*y*	*z*	*U*_iso_*/*U*_eq_	
Cl1	0.07984 (6)	0.10040 (17)	0.43221 (7)	0.0626 (5)	
N1	0.10533 (15)	0.4168 (4)	0.23756 (18)	0.0394 (9)	
H1C	0.1367	0.3550	0.2409	0.059*	
H1D	0.1195	0.4836	0.2706	0.059*	
H1E	0.0915	0.4628	0.1978	0.059*	
C14	0.0000	0.4263 (7)	0.2500	0.0401 (15)	
H14A	−0.0183	0.4888	0.2110	0.048*	
C13	0.0522 (2)	0.3310 (5)	0.2433 (3)	0.0489 (12)	
H13A	0.0693	0.2672	0.2820	0.059*	
H13B	0.0334	0.2691	0.2041	0.059*	
O1	0.19599 (14)	0.1670 (4)	0.26636 (16)	0.0522 (9)	
O2	0.14880 (16)	0.2723 (4)	0.13018 (17)	0.0610 (10)	
O6	0.22044 (16)	0.3852 (4)	0.37058 (16)	0.0600 (10)	
O5	0.15115 (18)	0.6523 (4)	0.3449 (2)	0.0699 (11)	
O3	0.07323 (17)	0.5386 (5)	0.10111 (19)	0.0694 (11)	
O4	0.10201 (18)	0.7507 (4)	0.2076 (2)	0.0748 (12)	
C11	0.2347 (2)	0.2291 (6)	0.3837 (3)	0.0613 (15)	
H11A	0.1988	0.1794	0.3863	0.074*	
H11B	0.2701	0.2174	0.4263	0.074*	
C12	0.2501 (2)	0.1626 (6)	0.3288 (3)	0.0593 (14)	
H12A	0.2846	0.2154	0.3246	0.071*	
H12B	0.2629	0.0612	0.3398	0.071*	
C2	0.1526 (3)	0.1189 (6)	0.1466 (3)	0.0683 (16)	
H2A	0.1580	0.0620	0.1117	0.082*	
H2B	0.1142	0.0871	0.1503	0.082*	
C1	0.2077 (3)	0.0951 (6)	0.2127 (3)	0.0634 (15)	
H1A	0.2155	−0.0089	0.2223	0.076*	
H1B	0.2449	0.1381	0.2102	0.076*	
C9	0.2026 (3)	0.6201 (8)	0.4103 (3)	0.0796 (19)	
H9A	0.1956	0.6718	0.4455	0.096*	
H9B	0.2419	0.6542	0.4096	0.096*	
C4	0.0927 (3)	0.4672 (8)	0.0535 (3)	0.080 (2)	
H4A	0.1335	0.5054	0.0591	0.096*	
H4B	0.0631	0.4888	0.0084	0.096*	
C3	0.0976 (3)	0.3036 (7)	0.0654 (3)	0.0751 (18)	
H3A	0.0581	0.2673	0.0648	0.090*	
H3B	0.1056	0.2548	0.0300	0.090*	
C6	0.0487 (3)	0.7661 (7)	0.1465 (4)	0.086 (2)	
H6A	0.0386	0.8695	0.1376	0.104*	
H6B	0.0127	0.7165	0.1488	0.104*	
C5	0.0641 (3)	0.6996 (7)	0.0914 (3)	0.085 (2)	
H5A	0.0303	0.7185	0.0482	0.102*	
H5B	0.1022	0.7436	0.0917	0.102*	
C8	0.1502 (4)	0.8053 (7)	0.3269 (4)	0.087 (2)	
H8A	0.1882	0.8290	0.3206	0.104*	
H8B	0.1483	0.8674	0.3622	0.104*	
C10	0.2064 (3)	0.4562 (7)	0.4218 (3)	0.0716 (17)	
H10A	0.2388	0.4350	0.4655	0.086*	
H10B	0.1665	0.4209	0.4205	0.086*	
C7	0.0943 (3)	0.8322 (6)	0.2623 (4)	0.083 (2)	
H7A	0.0571	0.7967	0.2673	0.099*	
H7B	0.0889	0.9362	0.2518	0.099*	
O9	0.0609 (3)	0.0673 (6)	0.3637 (2)	0.1150 (18)	
O8	0.0368 (2)	0.0512 (6)	0.4595 (3)	0.1106 (18)	
O10	0.0830 (5)	0.2559 (8)	0.4361 (5)	0.231 (5)	
O7	0.1380 (3)	0.0480 (16)	0.4675 (4)	0.257 (6)	

### Atomic displacement parameters (Å^2^)

**Table:**

	*U*^11^	*U*^22^	*U*^33^	*U*^12^	*U*^13^	*U*^23^
Cl1	0.0603 (8)	0.0785 (10)	0.0606 (8)	−0.0265 (7)	0.0363 (7)	−0.0224 (7)
N1	0.037 (2)	0.037 (2)	0.048 (2)	0.0041 (16)	0.0206 (17)	0.0043 (17)
C14	0.038 (3)	0.038 (4)	0.049 (4)	0.000	0.021 (3)	0.000
C13	0.040 (3)	0.040 (3)	0.071 (3)	−0.002 (2)	0.027 (2)	0.001 (2)
O1	0.0417 (18)	0.054 (2)	0.060 (2)	0.0123 (15)	0.0193 (17)	0.0100 (17)
O2	0.058 (2)	0.062 (2)	0.056 (2)	−0.0107 (17)	0.0150 (18)	−0.0049 (18)
O6	0.067 (2)	0.070 (2)	0.045 (2)	−0.0079 (18)	0.0247 (18)	0.0007 (18)
O5	0.076 (3)	0.052 (2)	0.091 (3)	−0.0118 (19)	0.044 (2)	−0.015 (2)
O3	0.068 (2)	0.074 (3)	0.055 (2)	−0.0096 (19)	0.0132 (19)	0.020 (2)
O4	0.057 (2)	0.059 (2)	0.104 (3)	0.0210 (19)	0.026 (2)	0.017 (2)
C11	0.052 (3)	0.065 (4)	0.058 (3)	−0.003 (3)	0.014 (3)	0.025 (3)
C12	0.043 (3)	0.055 (3)	0.070 (4)	0.003 (2)	0.013 (3)	0.020 (3)
C2	0.074 (4)	0.063 (4)	0.079 (4)	−0.008 (3)	0.042 (3)	−0.014 (3)
C1	0.071 (4)	0.046 (3)	0.089 (4)	0.012 (3)	0.049 (3)	−0.002 (3)
C9	0.087 (4)	0.101 (6)	0.056 (4)	−0.018 (4)	0.034 (4)	−0.025 (3)
C4	0.064 (4)	0.103 (5)	0.049 (3)	−0.027 (4)	−0.003 (3)	0.016 (3)
C3	0.072 (4)	0.095 (5)	0.044 (3)	−0.020 (3)	0.009 (3)	−0.013 (3)
C6	0.062 (4)	0.066 (4)	0.125 (6)	0.008 (3)	0.030 (4)	0.041 (4)
C5	0.059 (4)	0.076 (4)	0.084 (5)	−0.011 (3)	−0.009 (3)	0.053 (4)
C8	0.110 (5)	0.052 (4)	0.122 (6)	−0.004 (4)	0.071 (5)	−0.033 (4)
C10	0.070 (4)	0.093 (5)	0.054 (3)	−0.014 (3)	0.027 (3)	−0.005 (3)
C7	0.085 (4)	0.043 (3)	0.149 (7)	0.018 (3)	0.078 (5)	0.002 (4)
O9	0.159 (5)	0.123 (4)	0.065 (3)	0.033 (3)	0.045 (3)	0.005 (3)
O8	0.118 (4)	0.112 (4)	0.146 (4)	−0.048 (3)	0.100 (4)	−0.035 (3)
O10	0.412 (14)	0.111 (5)	0.310 (10)	−0.140 (7)	0.292 (11)	−0.112 (6)
O7	0.076 (4)	0.533 (19)	0.133 (6)	0.024 (7)	0.012 (4)	0.140 (9)

### Geometric parameters (Å, °)

**Table:**

Cl1—O7	1.332 (7)	C12—H12A	0.9599
Cl1—O9	1.399 (5)	C12—H12B	0.9600
Cl1—O10	1.403 (7)	C2—C1	1.500 (8)
Cl1—O8	1.410 (4)	C2—H2A	0.9600
N1—C13	1.492 (5)	C2—H2B	0.9601
N1—H1C	0.8900	C1—H1A	0.9599
N1—H1D	0.8900	C1—H1B	0.9600
N1—H1E	0.8900	C9—C10	1.493 (8)
C14—C13	1.528 (5)	C9—H9A	0.9600
C14—C13^i^	1.528 (5)	C9—H9B	0.9601
C14—H14A	0.9600	C4—C3	1.492 (8)
C13—H13A	0.9600	C4—H4A	0.9599
C13—H13B	0.9600	C4—H4B	0.9600
O1—C12	1.423 (6)	C3—H3A	0.9600
O1—C1	1.444 (6)	C3—H3B	0.9600
O2—C2	1.420 (6)	C6—C5	1.496 (9)
O2—C3	1.452 (6)	C6—H6A	0.9601
O6—C10	1.422 (6)	C6—H6B	0.9600
O6—C11	1.445 (6)	C5—H5A	0.9600
O5—C8	1.430 (7)	C5—H5B	0.9600
O5—C9	1.462 (7)	C8—C7	1.492 (9)
O3—C4	1.429 (8)	C8—H8A	0.9602
O3—C5	1.468 (7)	C8—H8B	0.9600
O4—C6	1.403 (7)	C10—H10A	0.9602
O4—C7	1.462 (7)	C10—H10B	0.9600
C11—C12	1.494 (7)	C7—H7A	0.9601
C11—H11A	0.9600	C7—H7B	0.9599
C11—H11B	0.9600		
			
O7—Cl1—O9	110.3 (5)	H1A—C1—H1B	108.3
O7—Cl1—O10	107.6 (7)	O5—C9—C10	109.1 (5)
O9—Cl1—O10	105.2 (5)	O5—C9—H9A	110.1
O7—Cl1—O8	111.1 (4)	C10—C9—H9A	111.4
O9—Cl1—O8	113.7 (3)	O5—C9—H9B	108.9
O10—Cl1—O8	108.5 (4)	C10—C9—H9B	109.0
C13—N1—H1C	109.5	H9A—C9—H9B	108.3
C13—N1—H1D	109.5	O3—C4—C3	110.2 (5)
H1C—N1—H1D	109.5	O3—C4—H4A	108.6
C13—N1—H1E	109.5	C3—C4—H4A	109.2
H1C—N1—H1E	109.5	O3—C4—H4B	110.0
H1D—N1—H1E	109.5	C3—C4—H4B	110.5
C13—C14—C13^i^	111.6 (5)	H4A—C4—H4B	108.3
C13—C14—H14A	109.3	O2—C3—C4	109.5 (4)
C13^i^—C14—H14A	109.2	O2—C3—H3A	110.1
N1—C13—C14	114.6 (4)	C4—C3—H3A	109.1
N1—C13—H13A	108.5	O2—C3—H3B	109.9
C14—C13—H13A	108.5	C4—C3—H3B	110.0
N1—C13—H13B	108.7	H3A—C3—H3B	108.3
C14—C13—H13B	108.5	O4—C6—C5	108.3 (5)
H13A—C13—H13B	107.8	O4—C6—H6A	109.6
C12—O1—C1	112.1 (4)	C5—C6—H6A	110.2
C2—O2—C3	112.0 (4)	O4—C6—H6B	110.6
C10—O6—C11	112.8 (4)	C5—C6—H6B	109.8
C8—O5—C9	111.8 (5)	H6A—C6—H6B	108.4
C4—O3—C5	114.2 (5)	O3—C5—C6	110.3 (5)
C6—O4—C7	111.7 (5)	O3—C5—H5A	109.2
O6—C11—C12	109.9 (4)	C6—C5—H5A	110.2
O6—C11—H11A	109.4	O3—C5—H5B	109.2
C12—C11—H11A	110.0	C6—C5—H5B	109.5
O6—C11—H11B	109.5	H5A—C5—H5B	108.3
C12—C11—H11B	109.8	O5—C8—C7	108.9 (5)
H11A—C11—H11B	108.2	O5—C8—H8A	109.8
O1—C12—C11	110.4 (4)	C7—C8—H8A	108.7
O1—C12—H12A	109.8	O5—C8—H8B	110.2
C11—C12—H12A	110.1	C7—C8—H8B	110.6
O1—C12—H12B	109.0	H8A—C8—H8B	108.6
C11—C12—H12B	109.2	O6—C10—C9	109.4 (5)
H12A—C12—H12B	108.2	O6—C10—H10A	110.4
O2—C2—C1	108.7 (4)	C9—C10—H10A	109.4
O2—C2—H2A	109.7	O6—C10—H10B	109.9
C1—C2—H2A	110.3	C9—C10—H10B	109.3
O2—C2—H2B	110.1	H10A—C10—H10B	108.5
C1—C2—H2B	109.4	O4—C7—C8	109.8 (4)
H2A—C2—H2B	108.6	O4—C7—H7A	109.0
O1—C1—C2	110.1 (4)	C8—C7—H7A	108.1
O1—C1—H1A	110.0	O4—C7—H7B	110.3
C2—C1—H1A	111.0	C8—C7—H7B	111.2
O1—C1—H1B	108.8	H7A—C7—H7B	108.3
C2—C1—H1B	108.6		
			
C13^i^—C14—C13—N1	−179.1 (5)	O3—C4—C3—O2	−66.2 (6)
C10—O6—C11—C12	−178.9 (4)	C7—O4—C6—C5	171.5 (5)
C1—O1—C12—C11	−176.3 (4)	C4—O3—C5—C6	−175.6 (5)
O6—C11—C12—O1	−64.8 (5)	O4—C6—C5—O3	65.9 (6)
C3—O2—C2—C1	178.0 (4)	C9—O5—C8—C7	−176.3 (5)
C12—O1—C1—C2	−173.0 (4)	C11—O6—C10—C9	171.0 (4)
O2—C2—C1—O1	66.2 (6)	O5—C9—C10—O6	61.6 (6)
C8—O5—C9—C10	−172.4 (5)	C6—O4—C7—C8	176.6 (5)
C5—O3—C4—C3	−177.2 (4)	O5—C8—C7—O4	−65.6 (6)
C2—O2—C3—C4	175.4 (5)		

Symmetry codes: (i) −*x*, *y*, −*z*+1/2.

### Hydrogen-bond geometry (Å, °)

**Table:**

*D*—H···*A*	*D*—H	H···*A*	*D*···*A*	*D*—H···*A*
N1—H1C···O1	0.89	2.10	2.958 (4)	161.
N1—H1E···O3	0.89	2.08	2.951 (5)	167.
N1—H1D···O5	0.89	2.12	3.007 (5)	177.
C10—H10B···O10	0.96	2.55	3.478 (13)	161
C13—H13A···O9	0.96	2.58	3.470 (7)	154
